# Do Two Screening Tools for Chikungunya Virus Infection that were Developed among Younger Population Work Equally as Well in Patients Aged over 65 Years?

**DOI:** 10.1371/journal.pntd.0005256

**Published:** 2017-01-05

**Authors:** Lidvine Godaert, Fatiha Najioullah, Lionel Bousquet, Thomas Malmontet, Benoît Fournet, Raymond Césaire, Jean-Luc Fanon, Moustapha Dramé

**Affiliations:** 1 Department of Geriatrics, University Hospitals of Martinique, Martinique, France; 2 Department of Virology, University Hospitals of Martinique, Martinique, France; 3 University of Reims Champagne-Ardenne, Faculty of Medicine, Reims, France; 4 University Hospital of Reims, Robert Debré Hospital, Department of Research and Public Health, Reims, France; University of Texas Medical Branch, UNITED STATES

## Abstract

**Background:**

Chikungunya is an endemo-epidemic infection, which is still considered as an emerging public health problem. The aim of this study was to evaluate in a 65+ population, the accuracy of two chikungunya screening scores that were developed in younger people.

**Methods:**

It was performed in the Martinique University Hospitals from retrospective cases. Patients were 65+, admitted to acute care units, for suspected Chikungunya virus infection (CVI) in 2014, with biological testing using Reverse Transcription Polymerase Chain Reaction. Mayotte tool and Reunion Island tool were also computed. Sensitivity, specificity, positive predictive value, negative predictive value, and Youden’s statistic were calculated.

**Results:**

In all, 687 patients were included, 68% with confirmed CVI, and 32% with laboratory-unconfirmed CVI. Fever (73.1%) and arthralgia (51.4%) were the most frequent symptoms. Sensitivity ranged from 6% (fever+headache) to 49% (fever+polyarthralgia); and Youden’s index ranged from 1% (fever + headache) to 30% (fever+polyarthralgia). PPV and NPV ranged from 70% to 95%, and from 32% to 43%, respectively.

**Conclusion:**

Performances were very poor for both tools, although specificity was good to excellent. Our results suggest that screening scores developed in young population are not accurate in identifying CVI in older people.

## Author Summary

Chikungunya virus is an alpha-virus transmitted by Aedes egyptii or albopictus bites. This infection is still considered as an emerging public health problem. In the acute stage of infection, typical physical signs of Chikungunya virus infection are febrile illness associated with severe and debilitating polyarthralgia affecting the small joints. Several studies have shown that mortality rates increased during the outbreak. Age over 85 years has been shown to be associated with increased mortality, and the mortality rate is higher in 65+ subjects than among younger population. During epidemics, prevalence rates vary from 18% to 48%. Rapid and reliable diagnosis is required especially for frail elderly population. Diagnosis based solely on physical examination may underestimate the magnitude of the epidemic. The systematic use of biological diagnosis during an outbreak is not feasible, especially in low- and middle-income countries. The use of predictive scores would thus be very helpful in this situation.

## Introduction

Chikungunya virus infection (CVI) is still considered as an emerging public health problem in both tropical and temperate regions [1]. It is usually symptomatic and may have three phases: acute (day (D)1 to D21), post-acute (D21 to D90), and chronic stage (beyond D90) [2, 3]; the latter two are sometimes absent. In the acute stage of infection, typical physical signs and symptoms of CVI are febrile illness associated with severe and debilitating polyarthralgia affecting the small joints. Severe functional disabilities characterise this phase. Other signs that can be observed include myalgia, headaches, or maculo-papular rash. In most cases, symptoms resolve within a few days with symptomatic treatment [2, 3].

Prior to the outbreak of 2005–2006 in Reunion Island (France), CVI was not considered to be life-threatening. Usually, the any cause overall mortality rate from CVI is considered to be low, comparable to that of seasonal influenza [4]. However, several studies have shown that mortality rates increased during the outbreak as compared to the same period in previous years [5–8]. Fatality increases in populations with atypical presentations, and the incidence of such atypical, serious or fatal cases increases with age. Indeed, age over 85 years has been shown to be associated with increased mortality [8], and the mortality rate is five times higher in subjects aged 65 years or older (65+) than among those under 45 years [5]. On Reunion Island, excess mortality concerned mainly people aged 75 years or older (75+) [9, 10]. Several comorbidities as well as increased age are linked with atypical presentation [11].

During epidemics, CVI prevalence rates are not fully known, and vary from 18% to 48% [12–14]. To meet patients’ needs, rapid and reliable diagnosis is required. Patients with CVI should be identified early, and receive appropriate care. Moreover, people with symptoms and signs consistent with CVI but who suffer from another type of disease must be diagnosed rapidly. Management without delay of differential diagnoses is essential. However, establishing a diagnosis of CVI in a simple and reliable way is very challenging. This concern is especially relevant to the frail elderly population. Furthermore, diagnosis based solely on physical examination may underestimate the magnitude of the epidemic [13].

The systematic use of biological diagnosis during an outbreak is not feasible, especially in low- and middle-income countries (e.g. due to lack of access to laboratory testing, difficulties processing samples, delays in the treatment of patients, etc.). The use of predictive scores would thus be very helpful in this situation. During the outbreak in Mayotte and Reunion Island, two predictive scores were developed. Sissoko et al. [15] retrospectively derived a clinical score (Mayotte tool) in a population of children and young adults. This score was based on the pairing of fever with the four most common clinical signs (polyarthralgia, myalgia, headaches, and back pain). More recently, Thiberville et al. [16] established a clinico-biological score (Reunion Island tool) from a population of patients aged 18 to 65 years. The performances of these scores were good, making them useful screening tools. However, they have not been evaluated in the elderly. Thus, we aimed to evaluate diagnostic performances of these two scores in a 65+ population, admitted to acute care units of Martinique University Hospital, with symptoms suggestive of CVI during the epidemic that occurred in 2014.

## Methods

### Study design and subjects

This was a diagnostic study performed in the University Hospital of Martinique (French West Indies) from retrospective cases.

Eligible patients were aged 65 years or older, admitted to acute care units including the emergency department (ED), for suspected CVI (presence of fever or arthralgia at admission based on Rajapakse et al 2010), from 10 January to 31 December 2014, and who underwent biological testing using Reverse Transcription Polymerase Chain Reaction (RT-PCR). Patients whose clinical and/or biological data were missing in their medical records, as well as those for whom it was not possible to compute either Mayotte tool or Reunion Island tool, were excluded.

### Data collection

We recorded baseline characteristics including age, sex, time since onset of Chikungunya symptoms, as well as presence or absence of the following features: fever, arthralgia (any of the following: knee, ankle, metacarpo-phalangeal joints, wrist, elbow, shoulder girdle, and pelvis), myalgia, digestive or neurological symptoms, and comorbidity burden (assessed using Charlson’s comorbidity index [17]). The Charlson’s comorbidity index measures patient comorbidity using the tenth International Classification of Diseases Diagnoses Codes. Each comorbidity has a weight (from 1 to 6) depending on its severity. The higher the score, the higher is the comorbidity burden. Biological testing included: white cells, neutrophils, lymphocytes, and RT-PCR. All patients included in this study had serum samples tested using RT-PCR with the RealStar^®^ Chikungunya RT-PCR Kit (Altona Diagnostics GmbH, Hamburg, Germany). We considered as confirmed CVI all suspected cases in whom biological confirmation was obtained by positive RT-PCR. The Mayotte tool and Reunion Island tool were calculated for all patients.

### Ethical considerations

The study was performed in accordance with the Declaration of Helsinki, and was approved by the “Commission Nationale de l’Informatique et des Libertés” (CNIL): authorisation number 1898399 v 0. Patient’s data was completely anonymised according to the CNIL requirements. All data was solely accessed and analysed retrospectively from the University Hospital of Martinique.

### Statistical analysis

The sample size was estimated based on the expected precision of sensitivity (Se) and specificity (Sp) confidence intervals. In a previous study [15], the prevalence of symptomatic CVI was 28% (318/1154). For an expected Se and Sp of 90% each, with a precision of 5%, and an alpha error of 5%, the estimated sample size was 192 for Se, and 494 for Sp. Therefore, we planned to include at least 494 patients.

In the acute phase, RT-PCR was considered as the gold standard to identify subjects with or without CVI. Sensitivity (%), specificity (%), positive predictive value (PPV, %), negative predictive value (NPV, %), and Youden’s index (J = Sensitivity (%) + Specificity (%)– 100) were estimated. Youden’s index is a single statistic that captures the performance of tests. Its value ranges from -100% (totally useless test) to 100% (perfect test).

Quantitative variables are described as mean ± standard deviation, and categorical variables as using number and percentage. Baseline characteristics were compared according to RT-PCR results using Student’s t-test (continuous variables) and chi^2^ test (categorical variables) Statistical analyses were performed using SAS release 9.4 (SAS Institute Inc., Cary, NC, USA).

## Results

**Fig 1:**
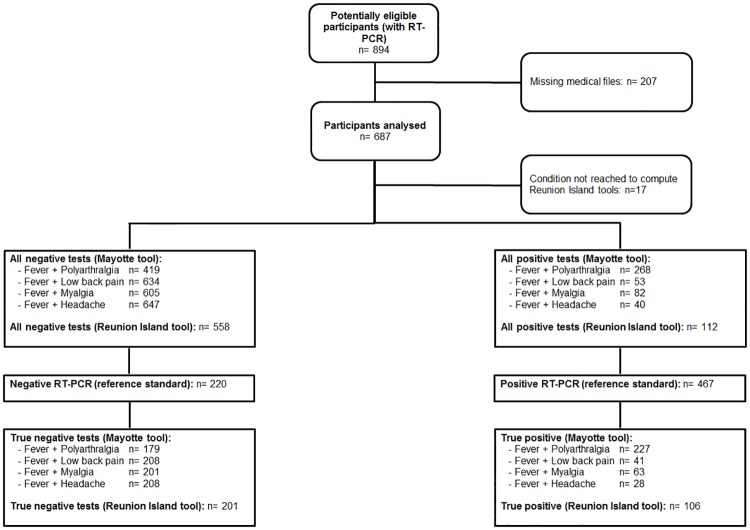
Flow chart describing the selection procedures of the subjects included in the study.

During the study period, 894 patients were potentially eligible. Among these, 207 were excluded. A flowchart of the study population is shown in Fig 1. Excluded subjects did not significantly differ from subjects included in terms of age (79.0±8.0 vs. 80.4±8.0 years, respectively) or sex (49% vs. 51% women, respectively). In all, 687 patients were considered in the present study. The mean Charlson’s comorbidity score was 1.7±1.9. The average time between onset of symptoms and admission was 1.3±2.3 days.

Clinical and biological characteristics at admission to hospital are presented in Table 1. Fever (73.1%) and arthralgia (51.4%) were the most frequent symptoms. The knee (22.3%), and the ankle (19.1%) were the most frequent sites of arthralgia. For biological characteristics, 77.9% of patients had a neutrophil count< 7500, and 61.3% had a lymphocyte count <1000.

**Table 1:** Clinical and biological characteristics at admission to hospital of subjects declaring symptoms of Chikungunya virus infection.

Symptoms or association of symptoms	Total N = 687		Chik+ n = 467		Chik- n = 220		
	n	%	n	%	n	%	p
Fever	502	73.1	371	79.4	131	59.6	<.0001
Arthralgia	353	51.4	292	62.5	61	27.7	<.0001
Arthralgia of the wrist	87	12.7	75	16.1	12	5.5	<.0001
Arthralgia of the metacarpophalangeal joints	47	6.8	40	8.6	7	3.2	.009
Arthralgia of the ankle	131	19.1	110	23.6	21	9.6	<.0001
Arthralgia of the knee	153	22.3	127	27.2	26	11.8	<.0001
Arthralgia of the shoulder	58	8.4	49	10.5	9	4.1	.005
Arthralgia of the pelvis	25	3.6	22	4.7	3	1.4	.03
Low back pain	69	10.0	48	10.3	21	9.6	.77
Myalgia	114	16.6	82	17.8	32	14.6	.32
Headache	46	6.7	31	6.6	15	6.8	.92
Digestive symptoms	151	22.0	91	19.5	60	27.3	.02
Neurological symptoms	130	18.9	88	18.8	42	19.1	.94
Fever + polyarthralgia	268	39.0	227	48.6	41	18.6	<.0001
Fever + low back pain	53	7.7	41	8.8	12	5.5	.13
Fever + myalgia	82	11.9	63	13.5	19	8.6	.07
Fever + headache	40	5.8	28	6.0	12	5.5	.78
Biological characteristics	m±SD		m±SD		m±SD		
White cell count	7608±4952		5986±2860		11052±6486		<.0001
Neutrophils	5744±4072		4501±2549		8507±5294		<.0001
Lymphocytes	1001±719		788±536		1476±838		<.0001

Missing data: white cells (n = 6); neutrophils (n = 23); lymphocytes (n = 23)

Chik+: laboratory-confirmed Chikungunya infection; Chik-: laboratory-unconfirmed Chikungunya infection

Patients with positive RT-PCR (chik+) CVI (n = 467) and patients with negative RT-PCR (chik-) CVI (n = 220) did not differ significantly with respect to age (80.6±7.8 versus 80.0±8.3, respectively; p = 0.33), sex (female sex 45.9% versus 52.9%, respectively; p = 0.09), or Charlson’s comorbidity score (1.6±1.8 versus 1.7±1.9, respectively; p = 0.73).

Performance indicators of the Mayotte tool and the Reunion Island tool are presented in Table 2. Sensitivity ranged from 6% (for fever+headache) to 49% (for fever+polyarthralgia). Youden’s index ranged from 1% (for fever+headache) to 30% (for fever+polyarthralgia). PPV and NPV ranged from 70% to 95%, and from 32% to 43%, respectively.

**Table 2:** Diagnostic performances of the Mayotte tool and the Reunion Island tool in the study population.

	TP n	FP n	TN n	FN n	Se (95% CI) %	Sp (95% CI) %	Youden’s index %	PPV (95% CI) %	NPV (95% CI) %
Mayotte tool									
Fever + polyarthralgia	227	41	179	240	49 (44–53)	81 (76–86)	30	85 (80–89)	43 (38–48)
Fever + low back pain	41	12	208	426	9 (7–12)	95 (91–97)	4	77 (64–87)	33 (29–37)
Fever + myalgia	63	19	201	404	13 (11–17)	91 (87–94)	4	77 (67–85)	33 (30–37)
Fever + headache	28	12	208	439	6 (4–9)	95 (91–97)	1	70 (55–82)	32 (29–36)
Reunion Island tool	106	6	210	348	23 (20–27)	97 (94–99)	20	95 (89–98)	38 (34–42)

TP: true positive; FP: False positive; TN: true negative; FN: False negative; Se: Sensitivity; Sp: Specificity; PPV: Positive Predictive Value; NPV: Negative Predictive Value; 95% CI: 95% confidence interval.

Youden’s index (%) = Sensitivity (%) + Specificity (%)– 100. A perfect test has a Youden’s index of 100%.

## Discussion

Our study shows that the diagnostic performance of two scores to screen for potential CVI, both developed in younger populations, is poor among older patients, as shown by the associated Youden’s index. While the specificity and the PPV of the scores are good to excellent, the sensitivity and NPV are mediocre, not to say poor. The specificity of the Mayotte tool [15] was 81% in our series, which was only slightly lower than the 89% reported in Sissoko’s seminal study. Regarding the Reunion Island tool [16], its specificity in our series was excellent, at 97%, compared to 85% in the original population. Conversely, the sensitivity of both scores was poor in our series; at 49% for the combination of fever plus polyarthralgia (for Mayotte tool), and 23% for Reunion Island tool. The authors of both these scores reported higher sensitivity (80% and 84% respectively). Using the clinical features score to compare three other pairs of symptoms found even lower sensitivity rates. These differences are likely due to the different clinical profiles observed in elderly subjects, which renders the use of scores developed in young populations perilous. In our study, the average age was 80.4 years, with an average comorbidity index of 1.7, underlining the geriatric profile of our population. In the two scores we tested, the average age in the development cohorts were 27.2±16.8 years for the Mayotte tool, and 40.1±12.4 years for the Reunion Island tool. Indeed, Mayotte tool is based on signs of fever plus polyarthralgia, which were present in 83.6% of the chik+ patients. In our series, this pair of symptoms was only observed in 48.6% of chik+ cases. This variation in the clinical profile of elderly subjects has previously been reported by other authors, who suggested that the incidence of atypical, severe or fatal cases increases with age [5]. In the Reunion Island tool developed by Thiberville et al. [16], the presence of fever and polyarthralgia were among the inclusion criteria, and therefore present in 100% of subjects. In our population, these two symptoms were found in 79.4% and 62.5% of chik+ patients, while we observed lymphopenia in 75.3% of chik+ subjects, compared to 79% in Thiberville’s study [16]. The symptom profile observed in our study was less specific, with fewer rheumatological symptoms than usually described in the semiology of CVI [3].

Modifications in clinical presentation in elderly people are frequently observed in general practice [18, 19]. In many cases, the primary complaint is rarely directly related to the precipitating event. This phenomenon has been widely studied, and led to the modelling of clinical presentations in elderly subjects by Fried et al. [19]. Fried’s diagnostic models take account of comorbidities, as well as the influence of functional and psychosocial factors. Indeed, the classical model in which symptoms correspond to those habitually observed in the causal disease is rarely the norm. Frequently, the physician (and/or the patient) may attribute recent symptoms to a known disease, whereas the symptoms may in fact be the result of an acute affection. Fried and colleagues called this the attribution model and facilitating complaint, whereby the concern identified at presentation to medical care was not the major underlying problem. In another model, termed the causal chain model, an elderly subject, often frail with multiple diseases, experiences an acute event that disturbs the patient’s fragile health equilibrium, and subsequently precipitates a chain of complications that may mask the initial events and/or aggravate co-existing diseases. All of these models illustrate the complexity of establishing an accurate diagnosis in this special population, especially using signs that were initially observed in a younger population.

Mediocre or poor sensitivity has major implications for the implementation of adequate treatment of CVI, even though treatment is mainly symptomatic. In older people, the problem is twofold. On one hand, sudden functional disability and loss of autonomy may lead to health complications (falls, dehydration, pressure ulcer, delirium, etc.). On the other hand, CVI may aggravate chronic disorders with possible adverse outcomes. In addition, older people may present atypical signs, which expose them to inadequate patient care due to serial misdiagnoses (differential diagnosis like dengue fever, leptospirosis, or bacterial infection).

The lack of validated tools for use in elderly patients is a common problem in routine care. Although a small number of screening tools or predictive scores have been validated for use in the elderly (e.g. the Mini Nutritional Assessment [20, 21], gait speed [22], or the timed “Up and Go” test [23]), many other instruments are widely used on a daily basis to aid management of elderly populations without robust scientific evidence confirming their clinimetric properties (e.g. the Wells score, or the Short Physical Performance Battery [24, 25].

Our study presents several strengths. Firstly, the sample size is very large, and includes specifically elderly patients (older age and higher comorbidity scores). The number of missing data per variable is also very low (3% at most). This provides a robust basis for results observed. The clinical and biological data were recorded by geriatric medicine and virology physicians from the hospital’s medical informatics system, with cross-checking from the patients’ medical records. Furthermore, confirmation of the diagnosis of CVI was obtained by RT-PCR using the same kits for all the subjects included in the study. Several limitations deserve to be addressed. We did not use serological testing to confirm CVI diagnosis. This could have impact in our results because people who have presented later their infection could have been misdiagnosed when using only RT-PCR. This would be very unlikely as patients for whom delay from onset symptoms to biological testing exceeded 48 hours were excluded from our study. The retrospective nature of the study could have been a limitation. Indeed, it would have been relevant to compare the performances of the Mayotte and Reunion tools in Martinique with the younger population they were developed in before comparing them in older population. Our population could be not representative of the overall elderly cases.

## Conclusion

The existing Mayotte tool and Reunion Island tool to predict CVI, developed in populations of younger patients, are not useful for the detection of CVI in 65+ patients. Population ageing and the likely recurrence of other epidemics of this virus justify the development of a specific clinical and/or clinico-biological score for elderly subjects in order to ensure early diagnosis and adequate management.

## Supporting Information

S1 Diagram STARD Diagram. (PDF)

S1 Checklist STARD Checklist. (DOCX)
