# Supplementary figures and images for: Do Two Screening Tools for Chikungunya Virus Infection that were Developed among Younger Population Work Equally as Well in Patients Aged over 65 Years?
Source: PLoS Negl Trop Dis. 2017 Jan 5;11(1):e0005256. doi: 10.1371/journal.pntd.0005256 (PMC5215818; doi:10.1371/journal.pntd.0005256)

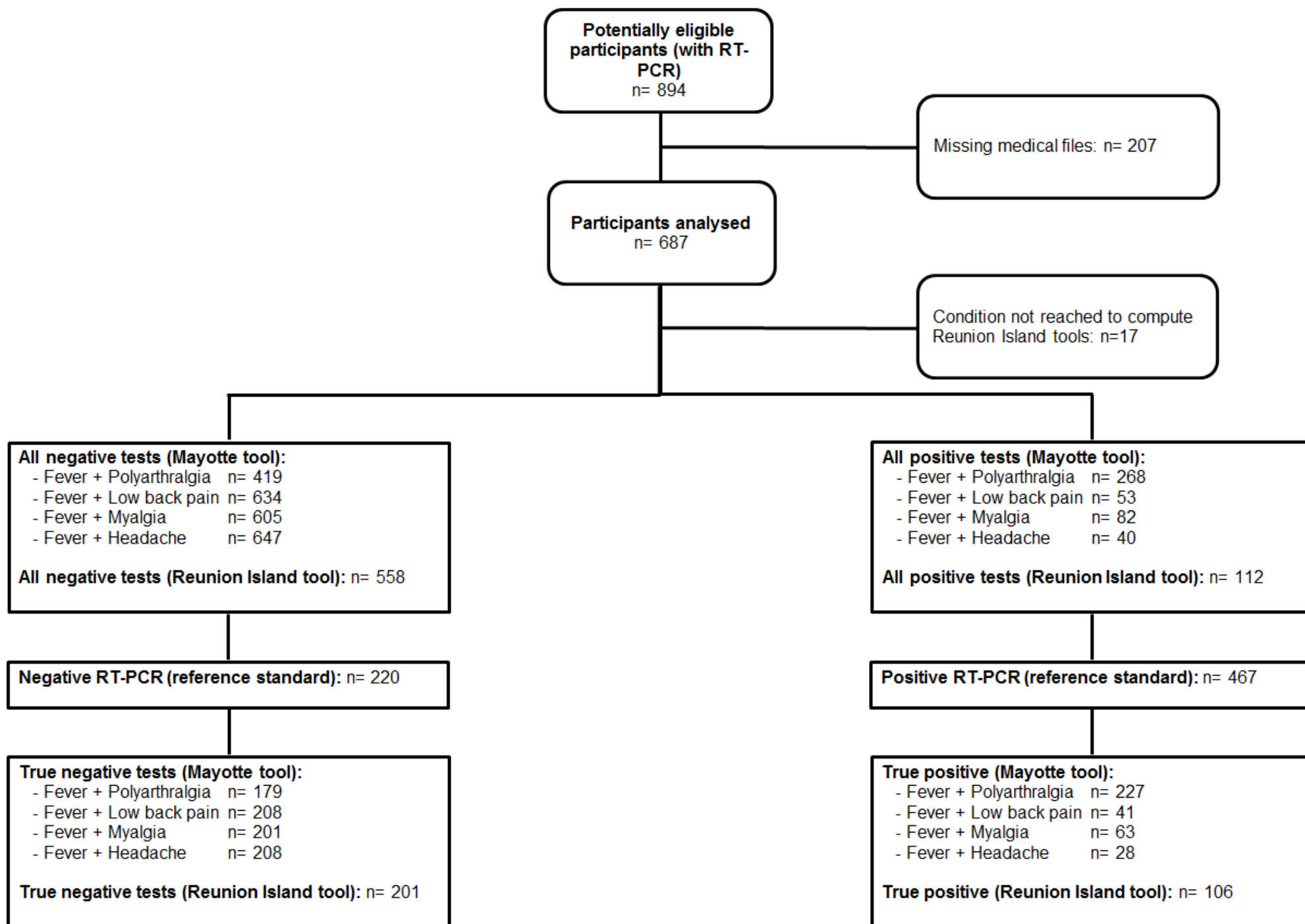

Supplement: S1 Diagram — (PDF) [file pntd.0005256.s001.pdf]
